# Ecdysteroid-Induced Programmed Cell Death Is Essential for Sex-Specific Wing Degeneration of the Wingless-Female Winter Moth

**DOI:** 10.1371/journal.pone.0089435

**Published:** 2014-02-18

**Authors:** Shuhei Niitsu, Kouhei Toga, Shigekazu Tomizuka, Kiyoto Maekawa, Ryuichiro Machida, Takehiko Kamito

**Affiliations:** 1 Department of Life Science, International Christian University, Tokyo, Japan; 2 Department of Biological Sciences, Tokyo Metropolitan University, Hachioji, Japan; 3 Graduate School of Science and Engineering, University of Toyama, Toyama, Japan; 4 Graduate School of Life and Environmental Sciences, University of Tsukuba, Tsukuba, Ibaraki, Japan; 5 Sugadaira Montane Research Center, University of Tsukuba, Sugadaira Kogen, Ueda, Nagano, Japan; University of Hyderabad, India

## Abstract

The winter moth, *Nyssiodes lefuarius*, has a unique life history in that adults appear during early spring after a long pupal diapause from summer to winter. The moth exhibits striking sexual dimorphism in wing form; males have functional wings of normal size, whereas females lack wings. We previously found that cell death of the pupal epithelium of females appears to display condensed chromatin within phagocytes. To provide additional detailed data for interpreting the role of cell death, we performed light microscopy, transmission electron microscopy, and TUNEL assay. We consequently detected two modes of cell death, i.e., dying cells showed both DNA fragmentation derived from epithelial nuclei and autophagic vacuole formation. To elucidate the switching mechanism of sex-specific wing degeneration in females of *N. lefuarius*, we tested the effects of the steroid hormone 20-hydroxyecdysone (20E) on pupal diapause termination and wing morphogenesis in both sexes. When 20E (5.4 µg) was injected into both sexes within 2 days of pupation, wing degeneration started 4 days after 20E injection in females, whereas wing morphogenesis and scale formation started 6 days after 20E injection in males. We discuss two important findings: (1) degeneration of the pupal wing epithelium of females was not only due to apoptosis and phagocytotic activation but also to autophagy and epithelial cell shrinkage; and (2) 20E terminated the summer diapause of pupae, and triggered selective programmed cell death only of the female-pupal wing epithelium in the wingless female winter moth.

## Introduction

The acquisition of wings is a key innovation that might enable the extreme diversification of insects. However, the secondary loss of flight ability has occurred independently in nearly all winged orders of insects [1]–[5]. One of the most interesting examples is the brachypterous or wingless lepidopteran insects. Certain species of moths have females that are described as wingless, whereas males of the same species have functional wings [6], [7]. Winglessness only in females is interesting in terms of a fitness trade-off between flight capability and reproduction. Such species provide good opportunities to investigate the developmental biology, physiology, ecology and molecular genetics underlying the regressive evolution of wing morphs.

Most winter-emerging geometrid moths have a unique life history that includes females having evolutionarily lost their wings due to seasonal adaptation of emergence during late autumn to early spring [8]. The winter geometrid moth, *Nyssiodes lefuarius*, exhibits striking sexual wing dimorphism. Males have wings that are functional, whereas the female wings are vestigial. Loss of wings is often accompanied by a loss of epidermal tissue. For example, degeneration occurs in minor workers of the ant *Pheidole megacephala* [9], and several moths [10], [11].

We previously described the developmental processes of wing reduction in *N. lefuarius* [12]. In this species, wing development is indistinguishable between the sexes until pupal development, as both sexes have complete and similarly sized pupal wing cases. During adult development, however, male wings transform into an adult structure whereas female wings are almost completely resorbed *via* cell shrinkage of the wing epithelial sheet and then develop into highly vestigial wings [12]. In spite of these dynamic morphological changes between the sexes, the cellular and physiological mechanisms causing female-specific winglessness are not sufficiently understood.

In insects, ecdysteroid hormones, primarily 20-hydroxyecdysone (20E), are the key steroids involved in the initiation of tissue differentiation during metamorphosis [13]. An earlier in vitro study reported that female-specific wing degeneration was induced by 20E [11]. The wingless-female tussock *Orgyia recens* moth studied by [11] undergo pupal-adult development just after pupation. In our study, however, the wingless-female winter moth *N. lefuarius* enters summer diapause just after pupation. Thus, the initiation of pupal-adult development is quite different between these two wingless-female moths. Many researchers have previously established the methods of 20E injection. Using this technique, it was possible to terminate diapause and to induce adult development in diapausing pupae of Lepidoptera [14]–[17]. However, little attention has been paid to in vivo effects of 20E on the termination of diapause and the induction of sexually dimorphic differentiation. It is possible that the developmental timing of female-specific wing degeneration in winter-emerging geometrid moths parallels the increase in ecdysteroid (20E) titer at the termination of summer diapause. We hypothesized that ecdysteroid terminates the summer diapause of pupae and triggers pupal-adult differentiation and programmed cell death (PCD) in the wings of female pupae in *N. lefuarius*. To break pupal summer diapause and to clarify what switching mechanism was triggering sex-specific degeneration of the wingless-female winter moth *N. lefuarius*, it is necessary to perform in vivo injection of 20E in this species.

Using light microscopy (LM), transmission electron microscopy (TEM), and cytohistochemistry (TUNEL assay), we undertook a detailed morphological and histological examination of the reduced pupal wings of females. We then injected various doses of ecdysteroid hormone (20E) into the newly molted diapause pupae of *N. lefuarius* to investigate whether the hormone is involved in such sex-specific wing degeneration by PCD.

## Results

### LM and TEM observations of pupal wings during metamorphosis

**Figure 1:**
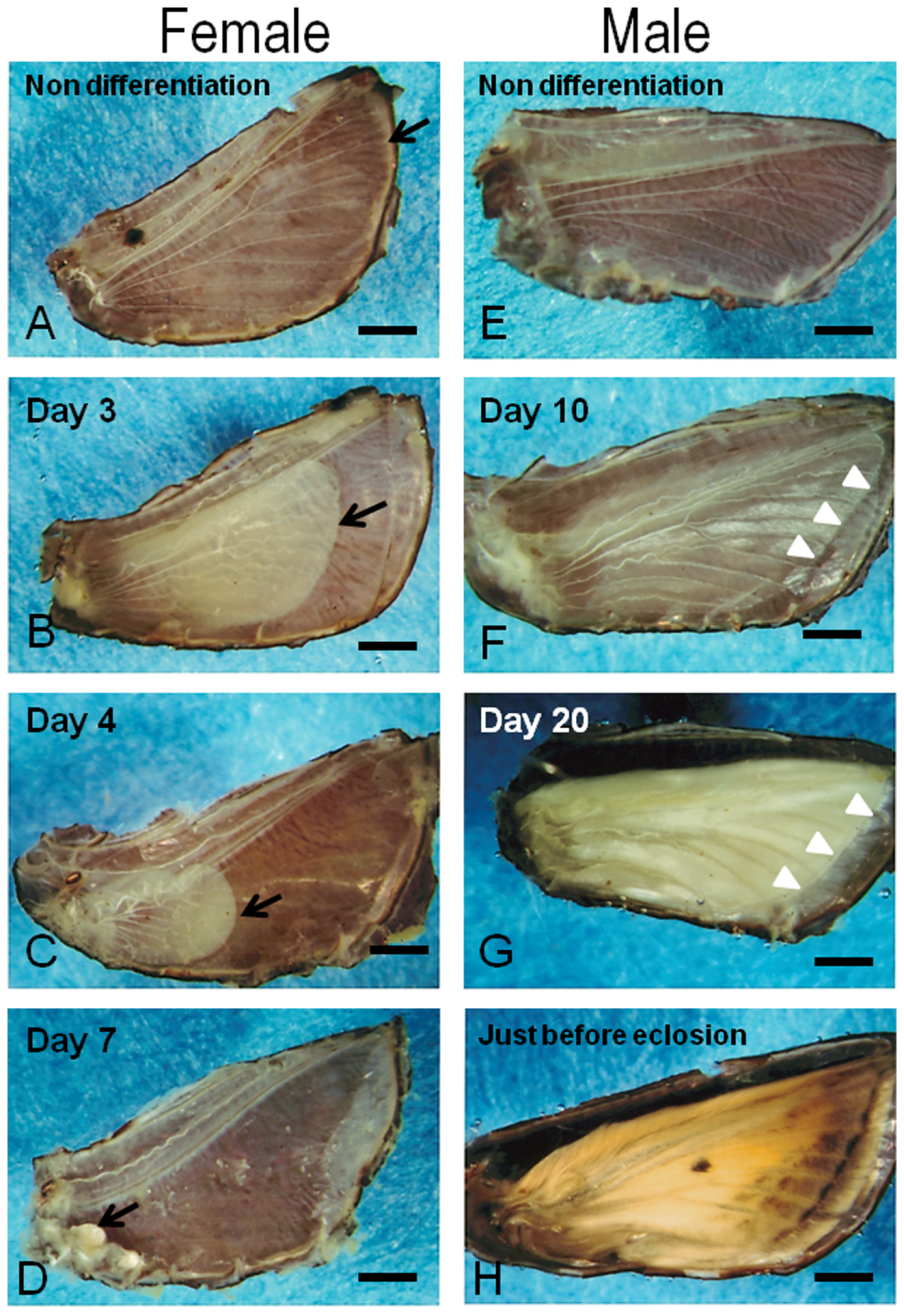
Developmental profiles of pupal wings in *Nyssiodes lefuarius*. Pupal wings of females (A–D) and males (E–H). (A–D) modified from [12]. In non-differentiation, the pattern of the pupal wing trachea in the female (A) is the same as that of the male (E) during the non-differentiation period. After differentiation, the pupal wing of females is degenerated. Note the significant pupal-wing degeneration on Day 3 (B), Day 4 (C) and Day 7 (D) after the beginning of pupal-adult development. By contrast, the pupal wing of males is formed normally on Day 10 (F) and Day 20 (G) after the beginning of pupal-adult development. (H) is just before adult eclosion. The arrow (A–D) points to the distal end of the degenerating wing of female. White arrowheads (F, G) indicate the position of the bordering lacuna (BL). Scale bar  = 1 mm.

**Figure 2:**
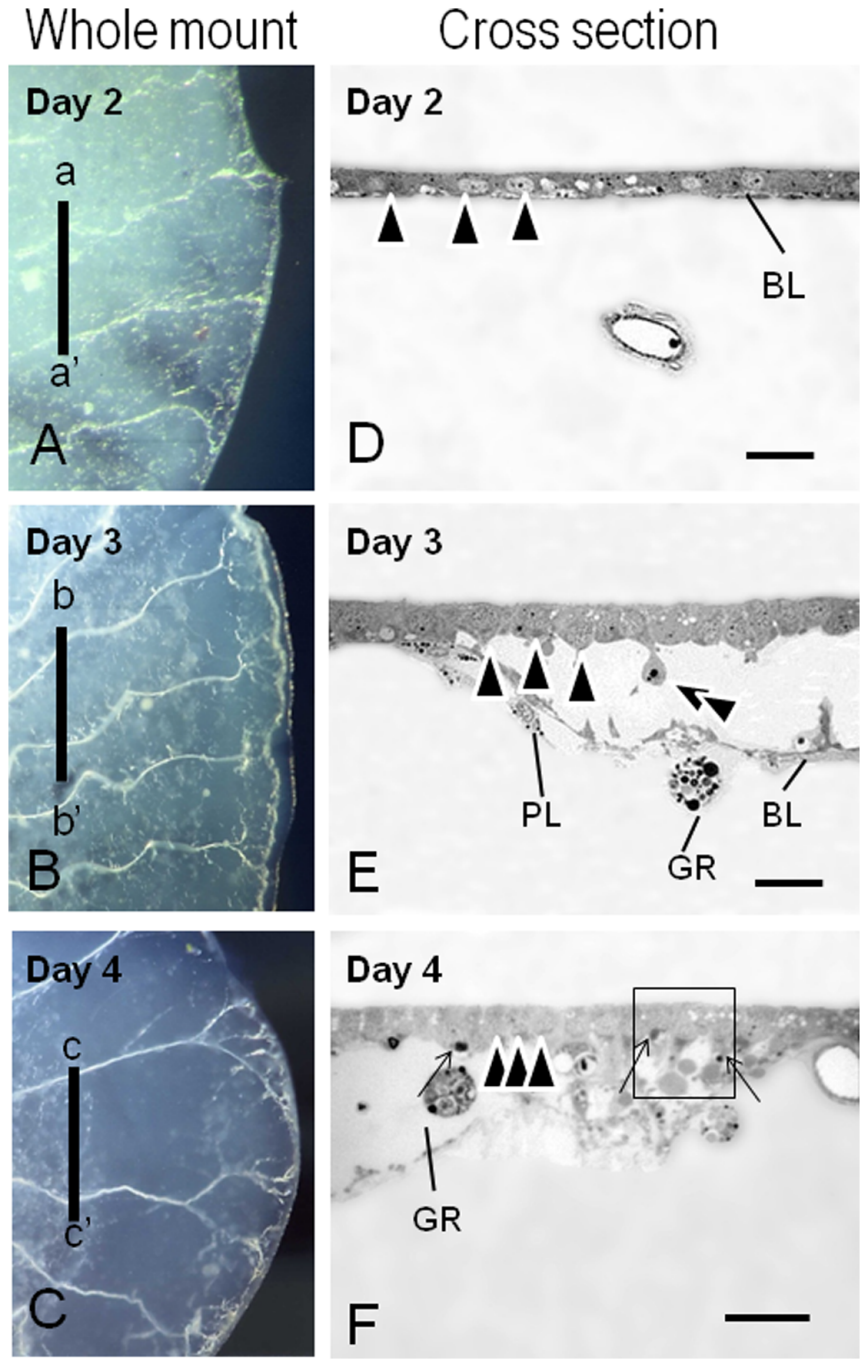
Whole mount images and cross sections of female pupal wings. Day 2 (A, D), Day 3 (B, E), and Day 4 (C, F) after the beginning of adult development. (A–C) Whole mount of female-pupal wings dissected from a pupa. (D–F) Cross sections of female-pupal wings. a-a′, b-b′, and c-c′ levels of the sections depicted in D–F. The box area in (F) corresponds to Fig. 3(B). An epithelial fragment was degenerating into the wing lumen (B, double arrowheads). An apoptotic body-like structure was also visible (F, black arrows). Note that the position of the epithelial nuclei shrank gradually (D–F, arrowheads). BL, basal lamina; PL, plasmatocytes; GR, granulocytes. Scale bar  = 20 µm.

**Figure 3:**
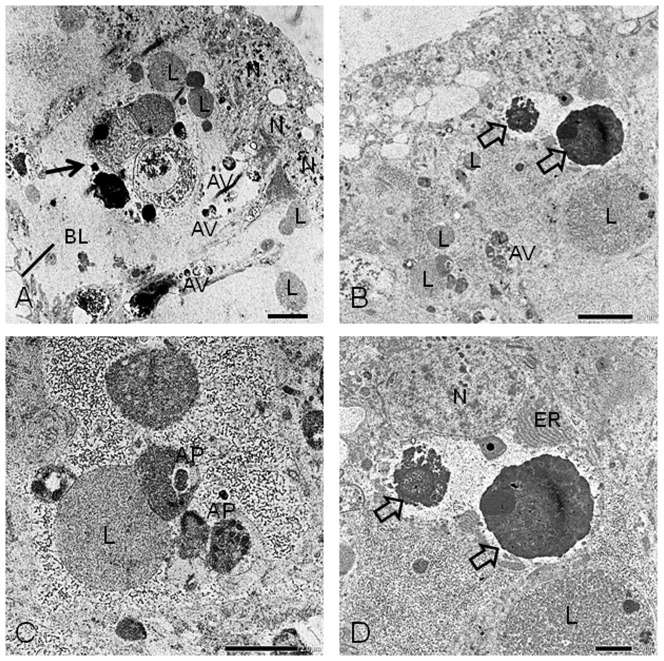
Transmission electron microscope micrographs of the pupal wing epithelium of females. Cell death of the pupal wing epithelium of females occurred strikingly on Day 4 after the beginning of pupal-adult development. (A) Phagocyte (black arrow) is engulfing fragmented forms of dead cells and lysosomes. (B) Condensed chromatin (open arrows) derived from nuclei are visible. (C) Autophagosome fuses to lysosomes to become an autolysosome. (D) Condensed chromatin and endoplasmic reticulum are visible. N, normal nuclei; ER, endoplasmic reticulum; BL, basal lamina; L, lysosome; AP, autophagosome; AV, autophagic vacuole. Scale bars: 5 µm for A–B and 2 µm for C–D.

In order to clarify the cellular changes of the pupal wings of females, observations by LM and TEM were performed. Epithelial cell shrinkage of the pupal wings in females occurred along the antero-posterior and proximo-distal axes (Fig. 1A–D). The process of female wing degeneration was complete by 7 days. During the pupal stage (Day 0 and Day 1), the wing epithelia of both sexes were attached to a thick pupal cuticle (Fig. 1A and E). On Day 2 after the beginning of pupal-adult development, the female wing cells began to retract from the pupal cuticle (Fig. 2A). At this stage, the wing epithelium consisted of an upper and lower monolayer. The inner surface of the wing epithelium was covered with basal lamina (Fig. 2D). No sign of pupal-adult development was observed. On Day 3 after the beginning of pupal-adult development, the pupal wing epithelium of females degenerated dramatically (Fig. 2B), possibly driven by the retraction of the basal lamina and the loss of cells from the epithelial monolayers (Fig. 2E). On Day 4 after the beginning of pupal-adult development, the female wing epithelium had degenerated further (Fig. 2C, F). Histological observations showed that wing degeneration of females was accompanied by epithelial cell shrinkage (Fig. 2D–F, arrowheads). By this stage, female wing length was about 3.5 mm, less than 1/3 of its original size, and small apoptotic-body like structures and many phagocytes were observed (Fig. 2F). Observation with TEM showed that phagocytes were engulfing lysosomes and cellular debris (Fig. 3A, arrow). Clumps of chromatin and fragmented forms of nuclei, which were a typical feature of apoptosis, were visible in the wing epithelia (Fig. 3B, D). A high magnification image showed that some autophagosomes had fused to lysosomes to become autolysosomes (Fig. 3C). Many autophagic vacuoles were frequently observed in the epithelia (Fig. 3A–C). These cellular events were a typical feature of autophagic cell death.

### Induction of sex-specific wing degeneration by 20E

**Figure 4:**
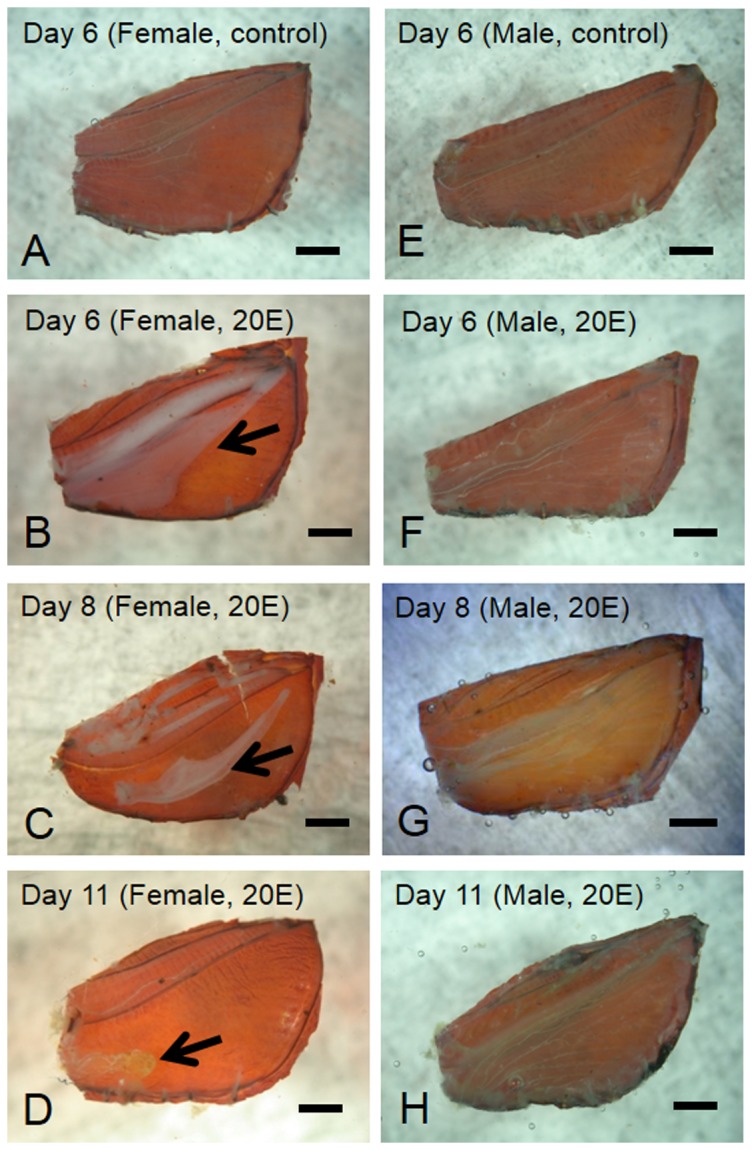
Morphological changes in pupal wings induced by injection of ecdysteroid (20-hydroxyecdysone, 20E). (A) Female-pupal wing without application of 20E on Day 6 after injection of 6 µl (4.7 µg) ethanol. Female-pupal wing on Day 6 (B), Day 8 (C), and Day 11 (D) after injection of 5.4 µg 20E. (E) Male-pupal wing after injection of 6 µl (4.7 µg) ethanol. Male-pupal wing on Day 6 (F), Day 8 (G), and Day 11 (H) after injection of 5.4 µg 20E. All wing epithelia are attached to their pupal-wing case. Arrows point the distal end of the degenerating wing epithelium in the female (B–D). Scale bar  = 1 mm.

To investigate whether 20-hydroxyecdysone (20E) is involved in sexual dimorphic formation and pupal wing degeneration, we injected pupae of *N. lefuarius* with 20E. Table 1 showed the effects of this 20E application on wing morphogenesis at Day 14. Application of 6 µl ethanol (control) of both sexes did not exhibit any morphological changes up to Day 14 (85% in males and 84% in females, See Table 1). Untreated pupae of both sexes did not exhibit any morphological changes up to Day 14 (100%; Fig. 4A, E). Injections of 1.8 µg 20E in both sexes also failed to induce adult differentiation (100%). The injection experiments using 5.4 µg 20E resulted in the formation of scale cells in male pupa (94.7%) and the degeneration of pupal wing epithelia of females (87.5%). Injection of an excessive dose of 16.2 µg 20E in male pupae triggered scale formation (64.5%), whereas injection of the dose in female pupae triggered wing degeneration (57.1%). Surprisingly, some of the females were not triggered to degenerate and to form wing sheet (14.3%). Some of the no regression females formed scale cells (11.4%). Taken together, female pupae receiving 16.2 µg 20E showed hyperecdysonism, or accelerated abnormal development.

**Table 1:** Induction ratio of 20E application by injection on wing morphogenesis at Day 14 in *Nyssiodes lefuarius*.

			Developmental events (%)				
Sex	Dose (µg)	Number	No sign	Regression	No regression		Dead
					Scale formation	Non scale formation	
Male	Untreated	25	100	0	0	0	0
	Control	25	84	0	0	0	16.0
	1.8	12	100	0	0	0	0
	5.4	19	11.1	0	94.7	0	5.3
	16.2	31	0	0	64.5	0	35.5
Female	Untreated	26	100	0	0	0	0
	Control	20	85	0	0	0	15.0
	1.8	10	100	0	0	0	0
	5.4	24	8.3	87.5	0	0	4.2
	16.2	35	2.9	57.1	11.4	2.9	25.7

**Figure 5:**
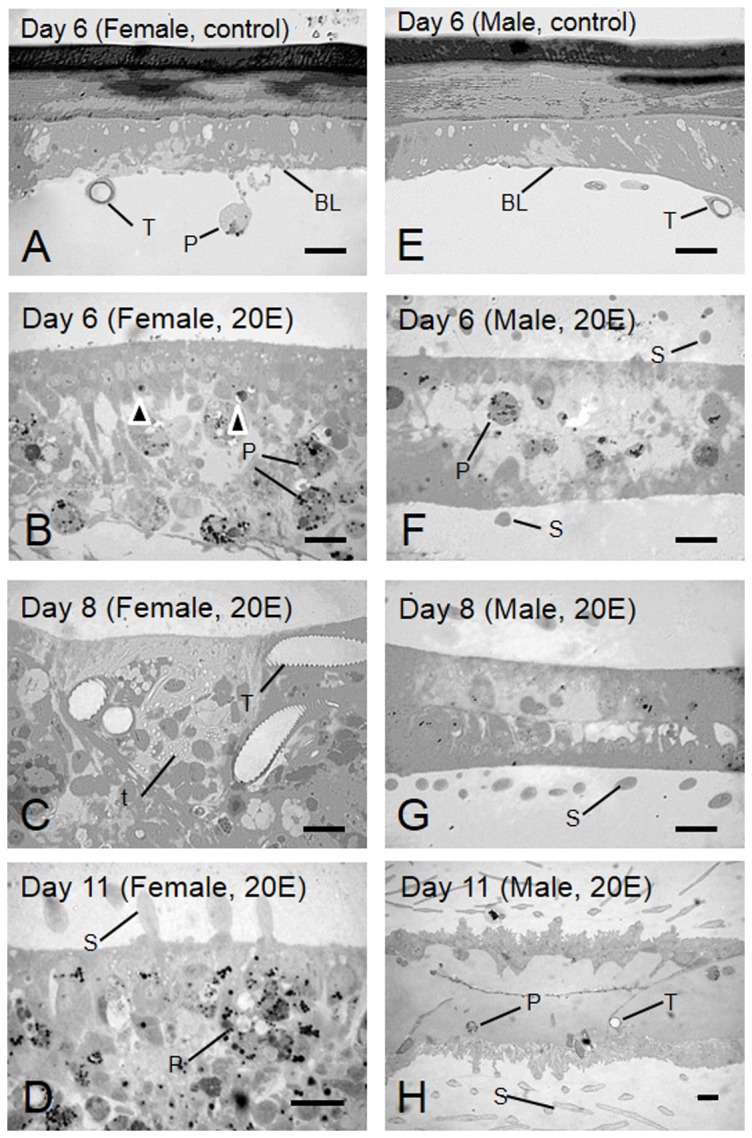
Cross sections of the pupal wing epithelium induced by injection of 20E. The pupal-wing epithelium of females (A–D) and males (E–H). Pupal wing epithelium of females (A) and males (E) on Day 6 after injection of 6 µl ethanol. Pupal-wing epithelium of females on Day 6 (B), Day 8 (C) and Day 11 (D) after injection of 5.4 µg 20E. Phagocytes (white arrows) and apoptotic-body-like structures (black arrows) are visible (D). Pupal-wing epithelium of males on Day 6 (F), Day 8 (G) and Day 11 (H) after injection of 5.4 µg 20E. Scale is visible in the pupal epithelium (F, G, H). BL, basal lamina; P, phagocyte; S, scale; T, trachea; t, tracheole. Scale bar  = 20 µm.

Next, we observed the morphological changes in female wings individually under injection of 20E (5. 4 µg). Up to Day 4 or 5 after injection, no developmental signs were observed in either sex. Developmental signs occurred on Day 5 or Day 6 after injection. There was wide variation in the termination of degeneration after injection of 5.4 µg 20E. To observe the effect of 20E on female-specific wing degeneration histologically, semi-thin sections of wings from injected pupae were prepared and then examined with LM. On Day 6, control pupae of both sexes had a small number of hemocytes and tracheae floating within the bi-layer of wing epithelial cells (Fig. 4A, E). Basal lamina attached to the wing epithelium (Fig. 5A, E). On day 6 after the injection of 20E to female pupae, the wing epithelia detached from the pupal cuticle. Wing size was reduced by half of their original size (Fig. 4B, 5B). At this stage, many hemocytes invaded the epithelial sheets, and caused phagocytosis in the pupal wing epithelia (Fig. 5B). Apoptotic-body-like structures were visible in the wing epithelia of females (Fig. 5B, black arrows). On Day 8 after injection of 20E to female pupae, wing size was reduced by seven tenths of their original size. (Fig. 4C). The wing trachea tightly coiled (Fig. 5C). On Day 11 after injection of 20E to female pupae, the degenerative events were complete (Fig. 4D). On Day 6 after injection of 20E to male pupae, the wing trachea migrated due to formation of wing vein (Fig. 4F). Scale formation began in this stage. Histological observation revealed that scale formation began and that basal lamina disappeared (Fig. 5F). On Day 8 after injection of 20E to male pupae (Fig. 4G), wing epithelia showed close contact (Fig. 5G). On Day 11 after injection of 20E to male pupae, wing epithelial sheet had thickened (Fig. 4H). Wing epithelia secreted new cuticle. The two monolayers separated and reformed basal lamina (Fig. 5H).

### Apoptosis in the pupal wings of females

**Figure 6:**
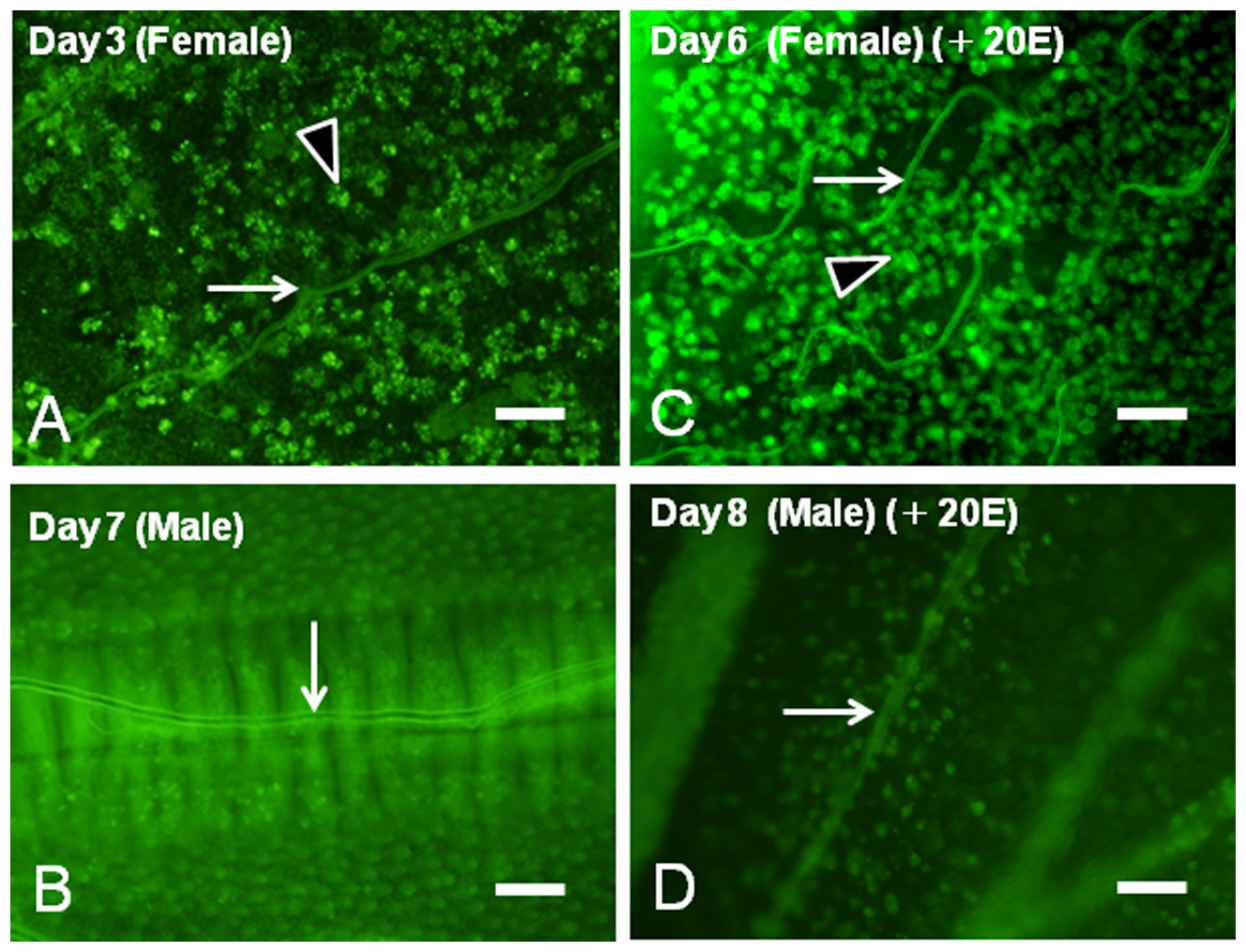
Acridine orange stain detecting the cell death of female pupal wings. (A) Whole mount of a female pupal wing on Day 4 after the beginning of pupal-adult development. (B) Whole mount of a male pupal wing on Day 7 after the beginning of pupal-adult development. (C) Whole mount of a female pupal wing on Day 6 after injection of 5.4 µg 20E. (C) Whole mount of a male pupal wing on Day 8 after injection of 5.4 µg 20E. Note that the coiling trachea synchronizes with wing degeneration (C, arrow). Most of the large signals in female wings appear to be fragmental nuclei trapped by the phagocytotic actions of hemocytes (A, C, arrowheads). White arrows (A–D) point to trachea. Scale bar  = 100 µm.

To further examine the progression of programmed cell death (PCD), we applied acridine orange staining to whole mounts of the pupal wing epithelia of males and females with or without 20E application. Female pupal wings on Day 4 after the beginning of pupal-adult development showed that spotted signals were detected in the pupal wing sheet (Fig. 6A). Male pupal wings on Day 7 after the beginning of pupal-adult development showed that small-spotted signals were detected (Fig. 6B). Most of the large signals in female wings appeared to be fragmented nuclei which were similar to those previously described in the wingless female tussock moth *O. recens* [11]. Female pupal wings on Day 6 after injection of 5.4 µg 20E showed that spotted signals were also visible (Fig. 6C). Male pupal wings on Day 8 after injection of 5.4 µg 20E showed that spotted signals which induced scale cells were detected (Fig. 6D).

**Figure 7:**
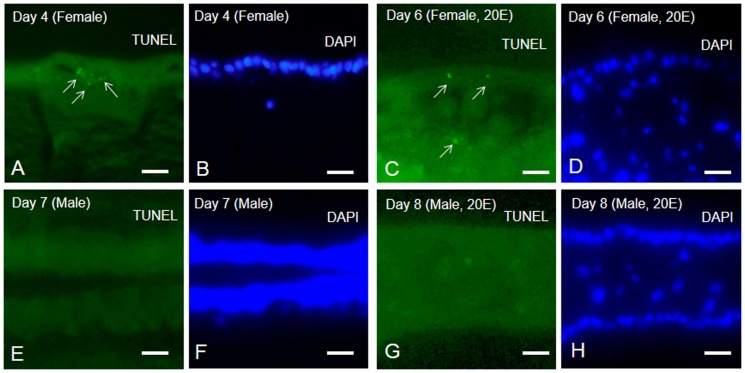
TUNEL and DAPI staining of cross sections in pupal wings. TUNEL assays (green: A, C, E, G) and DAPI staining (cyan: B, D, F, H) of cross sections of female and male pupal wings. (A) Cross section of female pupal wing on Day 4 after the beginning of pupal-adult development. (C) Cross section of female pupal wing on Day 6 after injection of 5.4 µg 20E. (E) Cross section of male pupal wing on Day 7 after beginning of pupal-adult development. (G) Cross section of male pupal wing on Day 8 after injection of 5.4 µg 20E. Note that TUNEL signals (light green) indicated by white arrows were visible in areas of the female wing epithelium. Scale bar  = 50 µm.

In cross-sections of the female pupal wings stained by the TUNEL method, we detected characteristic TUNEL-stained spotted signals, which were visible in the epithelium on Day 4 after the beginning of pupal-adult development (Fig. 7A). We also detected some TUNEL signals in the wing epithelium of females on Day 6 after injection of 5.4 µg 20E (Fig. 7C). Most of the TUNEL signals were visible in wing epithelial sheet (Fig. 7A, C. white arrows). In cross sections of male stained by the TUNEL method, we did not find any apoptotic signals (Fig. 7E–G).

## Discussion

Our previous studies showed that cell death of the pupal epithelium of females of *Nyssiodes lefuarius* appears to display condensed chromatin within the phagocytes [12]. In the present study, we performed light microscopy, transmission electron microscopy, acridine orange staining, and TUNEL assay. Our present study shows that the epithelial cells of females underwent PCD in an overlapping manner with apoptosis and autophagic cell death, i.e., the dying cells showed both condensation of chromatin derived from epithelial nuclei and autophagic vacuole formation characteristics typical of apoptotic and autophagic cell death. We also clarified that 20E terminates the summer diapause of pupae, triggers pupal-adult differentiation and then selective programmed cell death only in the female-pupal wing epithelium of the wingless female winter moth. A similar study in the female-wingless tussock moth *Orgyia recens* reported the tissue culture of pupal wings [11]. They showed a direct effect of 20E and the induction of selective cell death in vitro. However, their work was limited in terms of induction of apoptosis of the female pupal wings. Although our experimental results in vivo show a clear effect and the induction of sexually dimorphic differentiation; i.e., wing degeneration due to apoptosis in females, and differentiation of scale cells in males (Table 1), we acknowledge the possibility that the effect might be indirect in vivo. Several investigators have reported that the degeneration of wing tissues involves the process of apoptosis [9]–[12], [18]–[24]. Similar examples of autophagic cell death have been reported for female-specific wing degeneration in the wingless female tussock moth, *Orgyia leucostigma*, during pupal-adult development [10]. However, they did not describe the feature of apoptosis during wing degeneration in this species. In lepidopteran insects, PCD as a response to ecdysteroid is required for normal wing formation in order to determine final wing shape [19]. Although PCD also plays an important role in wing degeneration of some wingless female lepidopterans, and is triggered by ecdysteroid [11], [20]–[22], the developmental mechanism has not been studied from the standpoint of the genetic regulation of sexual wing dimorphism.

Programmed cell death (PCD) occurs during embryogenesis and metamorphosis and contributes effectively to organ resorption in the developing organs and tissues [25]–[30]. For example, PCD occurs during vertebrate limb formation [31], [32], and removes the amphibian tadpole tail during metamorphosis into a frog [33], [34]. Morphological studies of developing embryos resulted in the definition of three types of physiological cell death: apoptosis, autophagy and non-lysosomal [35], [36]. The results discussed here indicate that PCD in the female pupal wing has many characteristics in common with apoptosis and autophagic cell death in higher organisms. Even in dipteran insects such a phenomenon is seen during metamorphosis in tissues other than wings [37]. However, the interplay between apoptosis and autophagy is complex and not fully understood [38], [39].

In Lepidoptera, females with reduced wings are known in Geometridae, Lymantriidae, Noctuidae, Psychidae and several other families, and there have been some studies of these patterns of female-specific wing reduction [1]–[2], [40]–[41]. In *Orgyia leucostigma* (Lymantriidae), female wing discs are formed initially but are later destroyed by PCD during pupal-adult development [10]. In the congener *O. dubia*, the female pupa completely lacks pupal wing cases and the female adult is vermiform and apterous [42]. In yet another *Orgyia* species, *O. thyellina*, female wings show seasonal dimorphism. The autumn females have short wings whereas the summer females have normal wings [43], [44]. Wing degeneration has also been reported in females of several Psychidae moths. Adult females are usually wingless, but females of the presumably earliest lineages have normal wings. Bagworm moths may have secondarily lost their wings due to their case-dwelling life style. Several investigators have reported some ontogenetic and phylogenetic studies of wing degeneration in several species of wingless bagworm moths [45]–[47]. It has been hypothesized that the behavior of laying eggs in the larval case provides the selective advantage for the evolution of wing loss in female bagworm moths [47]. We propose that our findings of wing degeneration mechanisms may be utilized in other wingless female Lepidoptera. Our model insect, *Nyssiodes lefuarius*, belongs to the subfamily Ennominae in the family Geometridae [8]. The processes of wing degeneration observed in this study are probably a general phenomenon in geometrids, because all of the winter geometrid species possess a similar structure, i.e. female pupae possess well-developed wing cases [8].

In conclusion, this is the first report showing that female-specific wing degeneration of the pupal wing epithelium occurs by apoptosis and autophagy. We also show that 20E terminates the summer diapause of pupae, and triggers selective PCD in the wings of female pupae in *N. lefuarius*. Further studies of molecular analyses using next generation sequencing should advance our understanding of the molecular mechanisms of sexually dimorphic wing degeneration in the secondary loss of wings in insects.

## Materials and Methods

### Insect samples

Adult females of *Nyssiodes lefuarius* were collected on the banks of the Tamagawa River, Tokyo, Japan. We did not need to get specific permission to collect from this site, because the river is not part of a national park or sanctuary. Moreover, our study organism, *Nyssiodes lefuarius*, is a fairly common species throughout Japan. Consequently, we declare that our field studies did not involve an endangered or protected species and did not require specific permission. These females laid many eggs in the laboratory. Newly hatched larvae were reared under long-day photoperiod conditions (16L-8D) at 20°C with fresh leaves of *Artemisia* sp. Under these conditions, pupae entered summer diapause just after pupation. After 1.5 months, these pupae began to differentiate into pupal-adult development.

### Injections of 20-hydroxyecdysone into pupae

The appropriate concentration of 20E was based on the experiment of [14] in which the termination of pupal diapause of *Samia* pupal wings occurred at 5–20 µg 20-hydroxyecdysone (20E). 20E (Sigma) was dissolved in 99.5% ethanol. In the present study, the exact volume of 0.9 mg/ml of 20E was measured using a Hamilton microsyringe. Each of three doses of 20E, 1.8 µg (2 µl), 5.4 µg (6 µl), and 16.2 µg (18 µl) µg were injected into newly molted pupae (12–24 hours after pupation) of both sexes to achieve the desired concentration. Injections were done between the 5th and 6th abdominal segments. Negative controls were injected with 6 µl of 99.5% ethanol. No injected pupae were also prepared as untreated (Table 1).

### Preparation of wing tissues

Wing tissues were fixed in Karnovsky's fixative (2% paraformaldehyde +2.5% glutaraldehyde) and 1% osmium tetroxide. After dehydration through a series of ethanol and propylene oxide, the tissues were embedded in Epon 812 (TAAB). Semi-thin sections (1 µm thick) were cut using a UC6 ultramicrotome (Reica), mounted on microscopic slides, stained with Azur-B and then observed with a light microscope (LM).

For Transmission Electron Microscopy (TEM), pupal wings were sectioned (0.12 µm thick) with an ultramicrotome equipped with a diamond knife. Sections were stained with 4% uranium acetate for 20 min and then with 0.4% lead citrate for 10 min. These sections were observed under a LEM-2000 TEM (TOPCON, Japan) at 90 kV and JEM 1400 Plus electron microscope (JEOL, Japan) at 80 kV.

### Staining with acridine orange

Acridine orange staining was conducted according to [48] with minor modifications. Insects were dissected in phosphate-buffered saline (PBS) (137 mM NaCl, 2.7 mM KCl, 8.1 mM Na_2_HPO_4_, 1.47 mM KH_2_PO_4_). Dissected wings were incubated in a PBS+acridine orange solution at room temperature (20°C) in the dark for 5 min, washed in PBS for 10 min, and immediately examined and digitally photographed on a fluorescent light microscope (OLYMPUS BX 60, Tokyo, Japan).

### TUNEL assay

To investigate the possible occurrence of apoptosis, a terminal deoxynucleotidyl transferase mediated dUTP-biotin nick end labeling (TUNEL) assay [49] was performed using an *in situ* apoptosis detection kit (Takara, Kyoto, Japan). All samples were digested with a proteinase K solution (10 µg/ml proteinase K in PBS) for 15 min at room temperature to increase their permeability. After being washed twice with PBS for 5 min, these sections were incubated for 1 h with terminal deoxynucleotidyl transferase and fluorescein isothiocyanate-labeled dUTPs in a humidified chamber at 37°C to label the exposed 3′-hydroxyl ends of fragmented nuclear DNA. After terminating the reaction, the sections were again washed twice with PBS for 5 min. Sections were counterstained with 4, 6-Diamidino-2-phenylindole (DAPI; 30 nM solution in PBT) (Lonza, Walkersville, MD, USA) in the dark for 30 min at room temperature. The stained sections were mounted in Vectasheld (Vector Laboratories, Burlingame, CA, USA) and examined using a fluorescent light microscope (BZ-8100 Keyence, Osaka, Japan).
